# Pentoxifylline Regulates Plasminogen Activator Inhibitor-1 Expression and Protein Kinase A Phosphorylation in Radiation-Induced Lung Fibrosis

**DOI:** 10.1155/2017/1279280

**Published:** 2017-02-27

**Authors:** Jong-Geol Lee, Sehwan Shim, Min-Jung Kim, Jae Kyung Myung, Won-Suk Jang, Chang-Hwan Bae, Sun-Joo Lee, Kyeong Min Kim, Young-Woo Jin, Seung-Sook Lee, Sunhoo Park

**Affiliations:** ^1^Laboratory of Radiation Exposure & Therapeutics, National Radiation Emergency Medical Center, KIRAMS, Seoul, Republic of Korea; ^2^Department of Pathology, Korea Cancer Center Hospital, KIRAMS, Seoul, Republic of Korea; ^3^Molecular Imaging Research Center, KIRAMS, Seoul, Republic of Korea

## Abstract

*Purpose*. Radiation-induced lung fibrosis (RILF) is a serious late complication of radiotherapy. In vitro studies have demonstrated that pentoxifylline (PTX) has suppressing effects in extracellular matrix production in fibroblasts, while the antifibrotic action of PTX alone using clinical dose is yet unexplored.* Materials and Methods*. We used micro-computed tomography (micro-CT) and histopathological analysis to evaluate the antifibrotic effects of PTX in a rat model of RILF.* Results*. Micro-CT findings showed that lung density, volume loss, and mediastinal shift are significantly increased at 16 weeks after irradiation. Simultaneously, histological analysis demonstrated thickening of alveolar walls, destruction of alveolar structures, and excessive collagen deposition in the irradiated lung. PTX treatment effectively attenuated the fibrotic changes based on both micro-CT and histopathological analyses. Western analysis also revealed increased levels of plasminogen activator inhibitor- (PAI-) 1 and fibronectin (FN) and PTX treatment reduced expression of PAI-1 and FN by restoring protein kinase A (PKA) phosphorylation but not TGF-*β*/Smad in both irradiated lung tissues and epithelial cells.* Conclusions*. Our results demonstrate the antifibrotic effect of PTX on radiation-induced lung fibrosis and its effect on modulation of PKA and PAI-1 expression as possible antifibrotic mechanisms.

## 1. Introduction

In the clinic, radiation-induced lung fibrosis (RILF) is the main chronic adverse effect of radiotherapy [1–3]. But in spite of the urgent need to treat or mitigate RILF, there has been remarkably little progress in developing effective pharmacological or biological antifibrotic therapeutics [2, 4].

RILF is controlled by multiple regulatory molecules and among them, increased fibronectin (FN) is observed in both irradiated animals and cells [5, 6]. A recent study demonstrated that increased FN expression was significantly reduced by plasminogen activator- (PAI-) 1 inhibitor in human lung fibroblasts [7]. Knockout of the PAI-1 gene or knockdown of PAI-1 by small interfering RNA attenuates, whereas overexpression of PAI-1 enhances, fibrotic responses [7–10]. All these lines of evidence suggest that PAI-1 plays an essential role in the development of fibrosis.

Pentoxifylline (PTX) is already in clinical use for the treatment of peripheral vascular diseases [11, 12]. Recently, several in vitro studies have demonstrated that PTX inhibits fibroblast proliferation and extracellular matrix production [13–15]. Furthermore, PTX was found to lower plasma PAI-1 levels in obese patients [16]. These findings raise the possibility that PTX may have promise as an antifibrotic agent via regulation of PAI-1 in RILF. However, the used doses of PTX to produce these effects in vitro are high and might be extremely toxic [17]. Therefore, antifibrotic action of PTX alone using clinical dose remains to be demonstrated in vivo. For example, in a radiation-induced fibrosis rat and pig model no clinical or histological changes were observed with PTX treatment alone [18, 19]. However, the PTX dose was determined by a simple conversion based on body weight. Recent study suggested using the basis of a body surface area (BSA) normalization method for the conversion of the drug dosage from one species to another [20].

The purpose of this study was to answer two important questions in the field of RILF. First, we investigated whether changes in PAI-1 expression take place in the RILF rat model. Second, we tested whether PTX has any effect on the PAI-1 signaling during the reconstruction of RILF.

## 2. Materials and Methods

### 2.1. Animals

Male 6-week-old Wistar rats were obtained from Central Laboratory Animals (Seoul, Korea). The rats were kept under controlled conditions, with a constant temperature (23 ± 1°C) and photoperiod (12 hours of light, 12 hours of dark), and were allowed free access to regular chow and 3-stage filtered water. All animal experiments were approved by the Animal Investigation Committee of Korea Institute of Radiological & Medical Sciences (KIRAMS).

### 2.2. Irradiation Schedule and Pentoxifylline Treatment

Animals were divided into four groups: control, PTX, irradiation (IR), and IR + PTX. For hemithoracic irradiation, animals were anesthetized by intraperitoneal injection with tiletamine-zolazepam (30 mg/kg, Zoletil®; Virbac Korea, Seoul, Korea) plus xylazine hydrochloride (10 mg/kg, Rompun®; Bayer Korea, Seoul, Korea) and positioned on the bed. The right hemithorax was then ventrodorsally irradiated in a single exposure of 20 Gy at a dose rate of 2 Gy/min using an X-RAD 320 X-ray irradiator (Softex Korea, Goyang, Korea). Beginning 4 hours after irradiation, PTX (100 mg/kg/day; Huons, Jecheon, Korea) was intraperitoneally injected 5 days/week for the entire duration of the experimental period. The dose was the equivalent of that administered in clinical trials to patients receiving radiotherapy for lung cancer, determined according to a body surface area-based dose-translation formula [20].

### 2.3. Thoracic Micro-Computed Tomography (Micro-CT)

Scanning of the entire thorax was performed at 8, 12, and 16 weeks after irradiation using a commercial small-animal micro-CT scanner (Inveon; Siemens Healthcare, Knoxville, TN, USA). Micro-CT scan was performed under general anesthesia with 2% isoflurane (Forane®; ChoongWae Pharma, Seoul, Korea). Transverse CT images were captured and analyzed using ASIProVM software (Concorde Microsystems, Knoxville, TN, USA). To quantify fibrosis-related findings by micro-CT, 3 parameters (increased opacities, lung volume loss, and mediastinal shifting to the irradiated side) were analyzed as previously described [21].

### 2.4. Histopathological Examination of Lungs

Lung tissue samples were embedded in paraffin after fixation in 4% paraformaldehyde for 24 h, and 4 *µ*m sections were cut and stained with hematoxylin and eosin to assess structure and with Masson's Trichrome stain to visualize collagen deposition. The extent of fibrotic alteration was then quantified based on a modified Ashcroft scoring system [22].

### 2.5. Cell Culture and Treatment

The human lung epithelial cell lines BEAS-2B was purchased from the ATCC (Manassas, VA, USA). BEAS-2B cell lines were cultured in RPMI1640 supplemented with FBS (10%), penicillin (100 U/ml), and streptomycin (100 g/ml) in a 37°C humidified incubator with 5% CO_2_.

Cells were plated in 100 mm culture dishes at a density of 3 × 10^5^ cells per dish and irradiated at room temperature with a ^137^Cs Gamma cell 3000 Elan (Company Best Theratronics, Canada) at a dose rate of 3.25 Gy/minute for the time required to apply 5 Gy. 100 *μ*g/ml PTX was added in cell culture dishes at 30 minutes after irradiation. Cells were harvested 120 hours after radiation.

### 2.6. Western Blot Analysis

Lung tissues and cell lysates were prepared by extracting proteins with lysis buffer (40 mM Tris-HCl, pH 8.0, 120 mM NaCl, 0.1% Nonidet-P40) containing protease inhibitors and centrifuged at 14,000 ×g for 30 min. The supernatant was recovered and proteins were quantified using the Bio-Rad protein assay Kit. 10 *μ*g proteins were separated on sodium dodecyl sulphate- (SDS-) polyacrylamide gels and electrotransferred to a nitrocellulose membrane (Amersham, Arlington Heights, IL). The membrane was blocked with 5% nonfat dry milk in Tris-buffered saline and incubated with primary antibodies for overnight at 4°C. The membranes were then washed three times for 10 min in TBS-T and incubated with HRP-conjugated individual secondary antibody for 1.5 h at room temperature, followed by three 10 min washes in TBS-T. Finally, the signals were developed using the ECL reagent (GE Healthcare Bio-Sciences Corp., Piscataway, NJ, USA) using the manufacturer's protocol. Primary antibodies used in this study were antifibronectin (Abcam, Cambridge, UK); anti-TGF-*β*1 (Research Diagnostics, Flanders, NJ, USA); anti-phospho-Smad3, anti-Smad3, anti-phospho-PKA C, and anti-PKA C-*α* (all Cell Signaling Technologies, Danvers, MA, USA); anti-PAI-1 (BD Transduction Laboratories, San Jose, CA, USA); and *β*-actin (Santa Cruz Biotechnology, Santa Cruz, CA, USA). Densitometric analysis of band intensity was carried out using ImageJ software (National Institutes of Health).

### 2.7. Statistical Analysis

Data were expressed as the mean ± standard error of the mean (SEM). For analysis of differences between groups, one-way ANOVA followed by Student's* t*-test for individual comparisons among groups was performed. *P* < 0.05 was considered statistically significant.

## 3. Results

### 3.1. Assessment for RILF by Micro-CT and Histological Analysis

**Figure 1:**
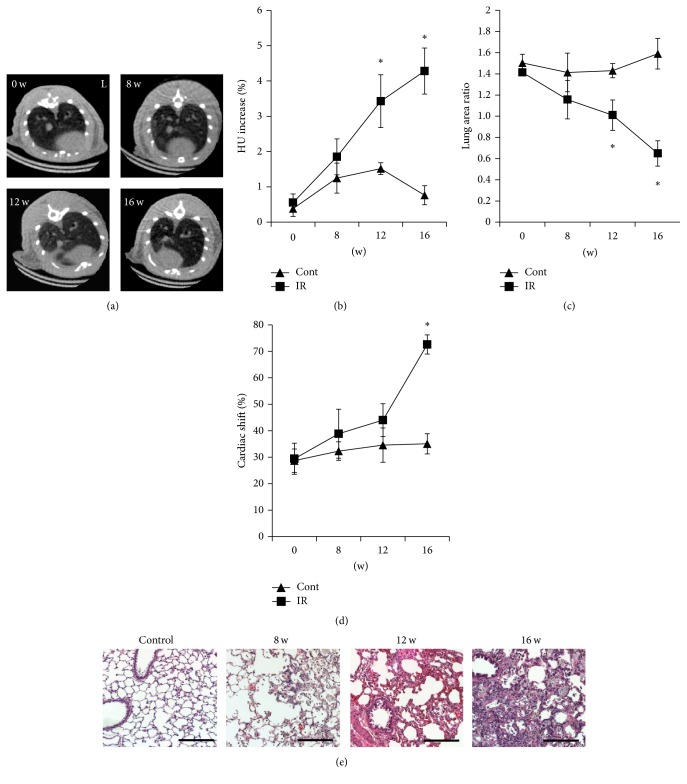
Evaluation of radiation-induced lung fibrosis. (a) Manifestation of RILF in nonirradiated or irradiated rats before irradiation (IR) or until 16 weeks after IR. Fibrosis-related findings such as increased lung density and volume loss were detected since 12 weeks after IR, and severe right deviation of heart was seen after 16 weeks of IR. (b–d) Lung density (b), lung area (c), and cardiac right-lateral shift (d) of control (Cont) and irradiated (IR) rats at the indicated times before (0 w) and after irradiation. Data are expressed as the mean ± standard error of the mean (SEM) (*n* = 6). ^*∗*^*P* < 0.05 versus control group. (e) Representative images of H&E stained sections of lung tissue of before (0 w) and after 8, 12, and 16 weeks after irradiation. Scale bar (right bottom) represents 200 *µ*m. Micro-CT, micro-computed tomography; RILF, radiation-induced lung fibrosis; HU, Hounsfield units; w, weeks.

Increased lung density and loss of volume were detected at 12 and 16 weeks after irradiation, and severe structural distortion of the heart and lung lobes appeared 16 weeks after irradiation (Figures 1(a)–1(d)). Histological sections showed macrophages infiltration on lung parenchyma at 8 weeks and interstitial septal thickening was progressed after 12 weeks. Extensive inflammatory cell infiltration, bronchial wall dilatation, and severe interstitial septal thickening were found at 16 weeks (Figure 1(e)). On the basis of these findings, 16 weeks after irradiation was selected as an appropriate time point to investigate RILF in further experiments.

### 3.2. Effect of PTX Administration on RILF

**Figure 2:**
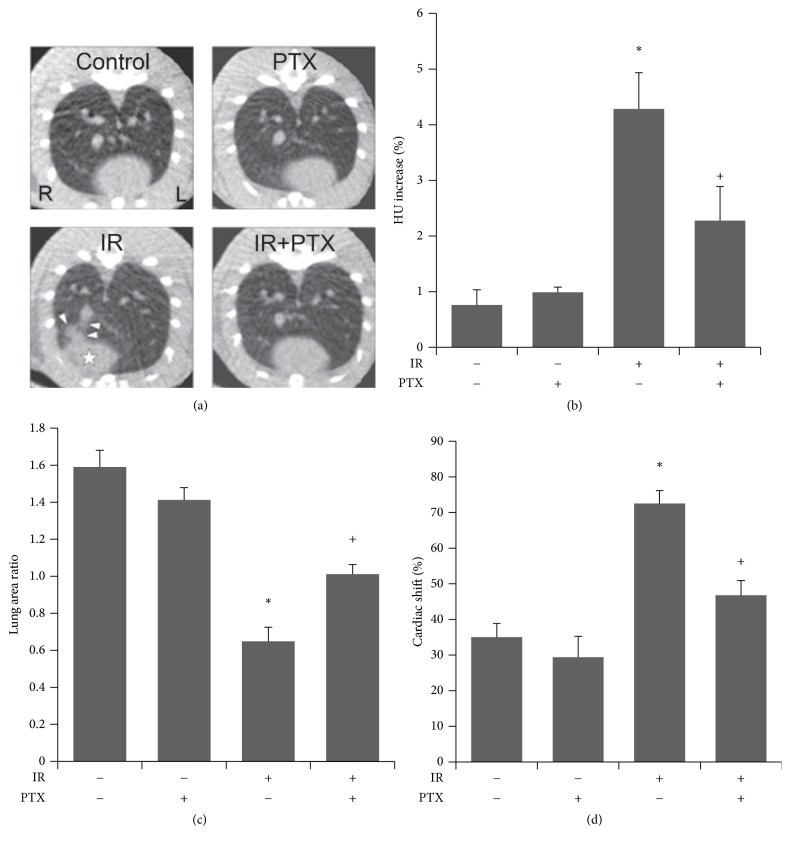
Micro-CT analysis of effect of PTX on RILF. (a) Representative micro-CT images of irradiated or nonirradiated rats with or without PTX administration. PTX administration ameliorated the fibrotic changes including increased lung density (white arrowheads), decreased lung volume, and structural distortion of heart (white star) and lung lobes in irradiated rats. (b–d) Lung density (b), lung area (c), and cardiac right-lateral shift (d) of control (Cont) and irradiated (IR) rats at 16 weeks after irradiation. Data are expressed as the mean ± SEM (*n* = 6). ^*∗*^*P* < 0.05 versus control group; ^+^*P* < 0.05 versus IR group. Micro-CT, micro-computed tomography; PTX, pentoxifylline; RILF, radiation-induced lung fibrosis; HU, Hounsfield units.

At 16 weeks after irradiation, micro-CT revealed that PTX ameliorated the fibrotic findings (Figure 2(a)). Quantification of micro-CT data showed that irradiation significantly increased lung density in the IR group compared to the control group, and PTX treatment after irradiation significantly attenuated this effect (Figure 2(b)). The ratio of right lung area to that of left lung was significantly reduced in the IR group compared to the control group, and PTX treatment following irradiation significantly alleviated this loss in volume compared with the group treated with irradiation alone (Figure 2(c)). Moreover, hemithoracic irradiation significantly induced cardiac right-lateral shift compared to the control group, and PTX administration after irradiation significantly decreased this shift compared to the group treated with irradiation alone (Figure 2(d)).

### 3.3. Effect of PTX on (Irradiation-Induced) Histopathological Changes and ECM Deposition In Vivo

**Figure 3:**
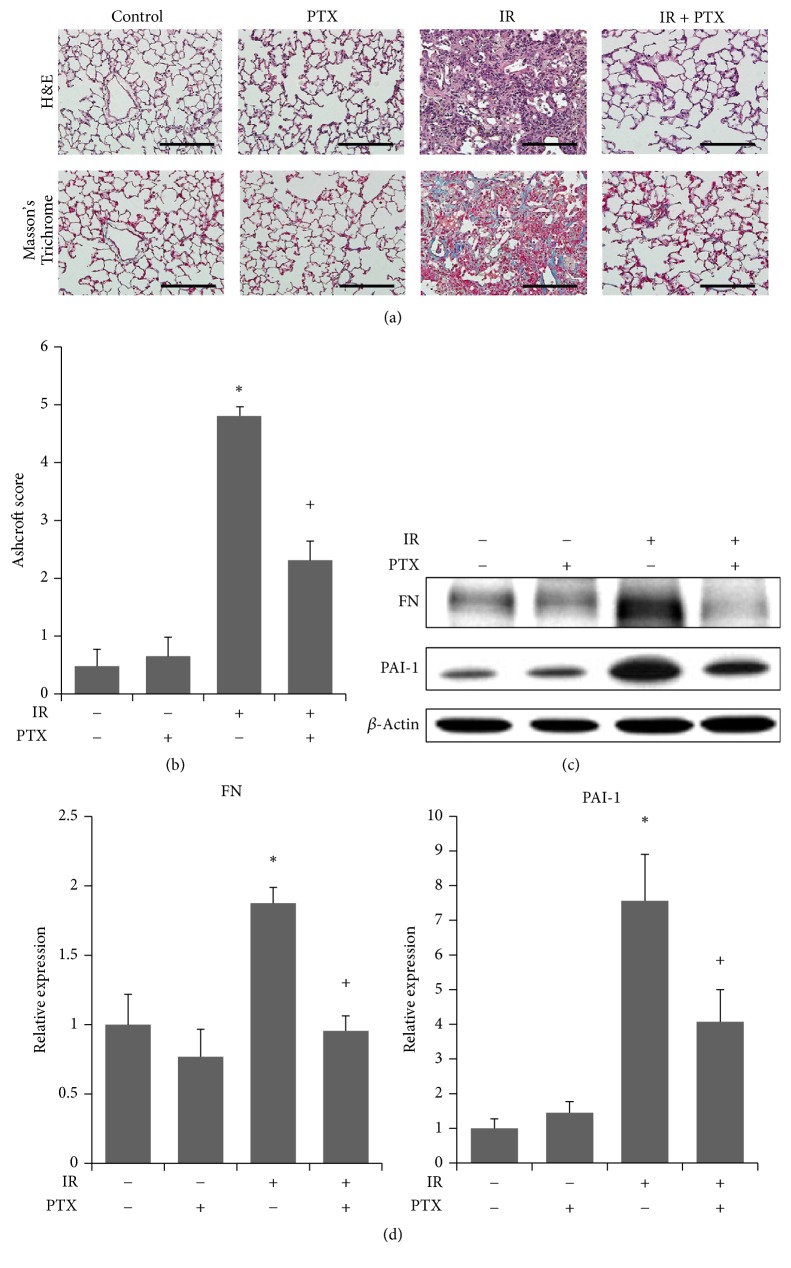
Histological finding and assessment of ECM deposition. (a) Representative images of H&E (upper) and Masson's Trichrome (lower) stained sections of lung tissue of control (Cont), PTX-treated (PTX), irradiated (IR), and irradiated and PTX-treated rats 16 weeks after irradiation. Scale bars represent 200 *µ*m. (b) Quantification of lung fibrosis in the four treatment groups in (a) expressed as modified Ashcroft score. Data are expressed as the mean ± SEM (*n* = 6). ^*∗*^*P* < 0.05 versus control group; ^+^*P* < 0.05 versus IR group. (c) Western analysis and (d) quantification of FN and PAI-1 expression in lungs of rats in the four indicated treatment groups. Band densities were normalized to those of *β*-actin. Data were normalized to control and expressed as the mean ± SEM (*n* = 6). ^*∗*^*P* < 0.05 versus control group; ^+^*P* < 0.05 versus IR group. H&E, hematoxylin and eosin staining; PTX, pentoxifylline; FN, fibronectin; PAI-1, plasminogen activator inhibitor-1.

Histological analysis demonstrated that irradiation induced thickening of alveolar walls, expansion of alveolar spaces, destruction of normal alveolar structures, and excessive collagen deposition in irradiated right lung lobes at 16 weeks after irradiation. All of these effects were ameliorated by PTX treatment after irradiation (Figure 3(a)). Moreover, quantification using a modified Ashcroft score revealed that irradiation significantly increased fibrotic changes compared to the control group (Figure 3(b)), and rats treated with PTX after irradiation exhibited significantly decreased fibrosis in comparison to the IR group.

Expressions of FN and PAI-1 were significantly increased, molecular indicators of fibrotic change, by irradiation compared to the control group, and PTX significantly reduced expression levels of both proteins compared to the group treated with irradiation alone (Figures 3(c) and 3(d)).

### 3.4. Effect of PTX on (Irradiation-Induced) Changes of Signaling Proteins In Vivo

**Figure 4:**
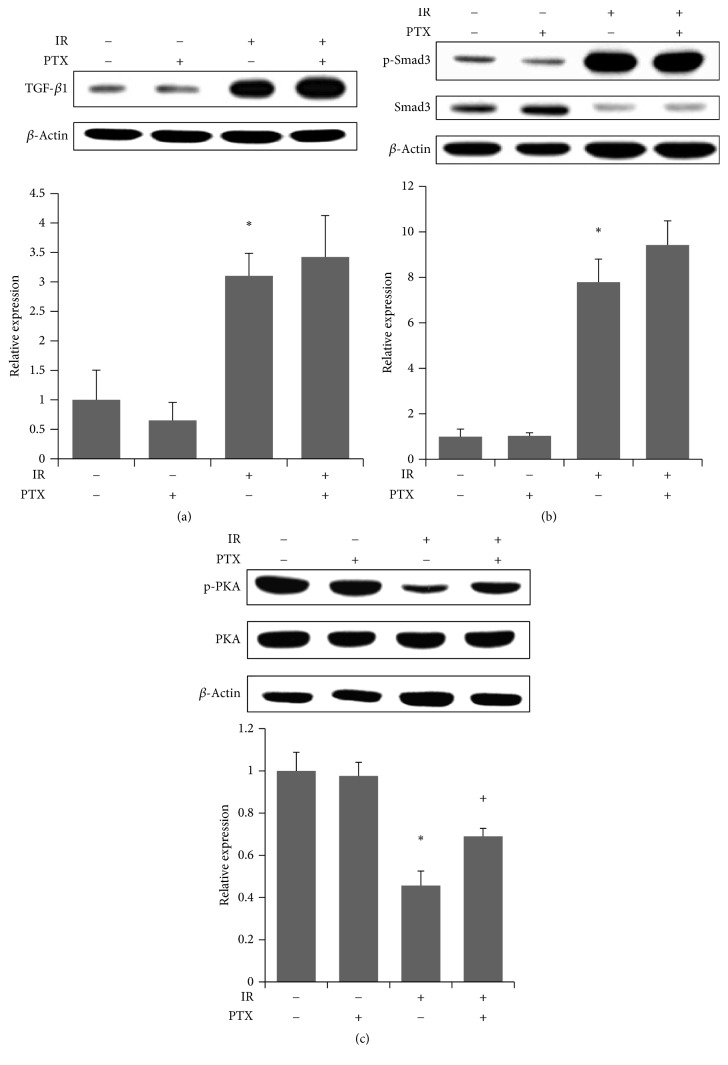
Western analysis of signaling proteins upstream of PAI-1. (a–c) Western analysis (upper) of (a) TGF-*β*, (b) total and phosphorylated Smad3, and (c) total and phosphorylated PKA and quantitation (lower) in lungs at 16 weeks after irradiation. Band densities were normalized to those of *β*-actin. Values are expressed relative to control and are the mean ± SEM (*n* = 6). ^*∗*^*P* < 0.05 versus control group; ^+^*P* < 0.05 versus IR group. PAI-1, plasminogen activator inhibitor-1; TGF-*β*, transforming growth factor-*β*; PKA, protein kinase A; PTX, pentoxifylline.

To examine the effect of irradiation on the signaling pathway upstream of PAI-1, both TGF-*β*/Smad and PKA were analyzed (Figure 4). Irradiated lungs showed significant increases in TGF-*β*1 expression and Smad3 phosphorylation and decreased PKA phosphorylation at 16 weeks after irradiation compared with the control group. Whereas PTX administration partially restored the attenuated phosphorylation of PKA (Figure 4(c)), no effect was observed on levels of TGF-*β*1 and phosphorylated Smad3 compared to the IR group (Figures 4(a) and 4(b)).

### 3.5. Effects of PTX on PAI-1 Related Signaling in Lung Epithelial Cells

**Figure 5:**
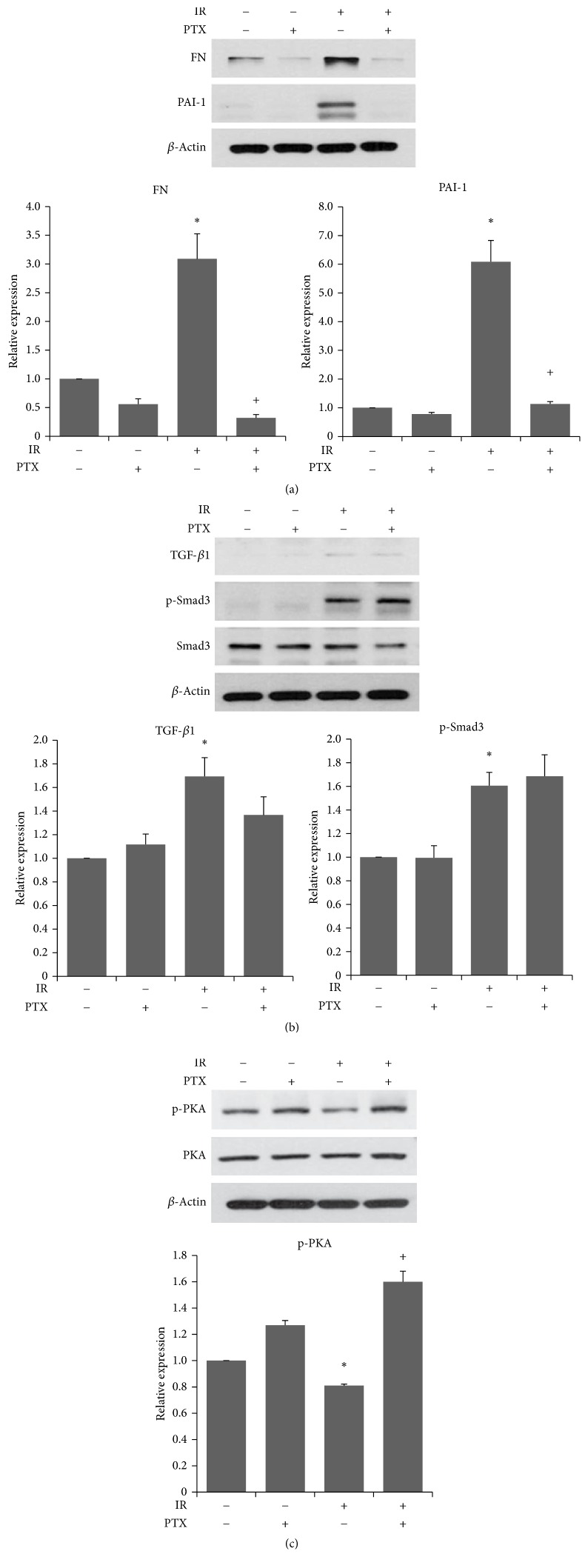
Western analysis of PAI-1 signaling related proteins in lung epithelial cells. (a–c) Western analysis of FN and PAI-1 expression (a), TGF-*β*/Smad3 (b), and PKA (c) in BEAS-2B cells at 120 hours after irradiation. Band densities were normalized to those of *β*-actin. Values are expressed relative to control and are the mean ± SEM. ^*∗*^*P* < 0.05 versus control group; ^+^*P* < 0.05 versus IR group. Each experiment was repeated at least three times.

After 5 Gy irradiation, expressions of FN and PAI-1 were increased and PTX treatment reduced significantly expression levels of both proteins in BEAS-2B cells (Figure 5(a)). And irradiated cells expressed significant increases in TGF-*β*1 expression and Smad3 phosphorylation (Figure 5(b)) and decreased PKA phosphorylation (Figure 5(c)). We also demonstrated antifibrotic potential of PTX by modulating PKA, and no effect was observed on levels of TGF-*β*1 and phosphorylated Smad3 (Figures 5(b) and 5(c)). These results were consistent with in vivo results.

## 4. Discussion

In this study, we used micro-CT and histopathological analyses to demonstrate that PTX attenuated RILF and explored possible target molecules involved in its antifibrotic effects. Lung density and area and cardiac right-lateral shift evaluated by micro-CT and histopathological analyses showed significant radiation-induced changes at 16 weeks after irradiation. Quantification of fibrosis revealed that irradiation significantly induced lung fibrosis. However, PTX treatment after irradiation ameliorated changes in all parameters and resulted in significantly reduced fibrosis.

Expressions of PAI-1 and FN were significantly increased by irradiation, and PTX significantly reduced expression levels of both proteins. PAI-1 expression is elevated under pathologic fibrotic conditions, which contributes to a reduced rate of fibrinolysis and subsequent decrease in degradation of ECM components, including FN, resulting in tissue fibrogenesis [22]. Elevated PAI-1 level also appears to be critical to the development of lung fibrosis [7–10]. Therefore, our results indicate that the antifibrotic effect of PTX may be mediated through downregulation of PAI-1.

TGF-*β*/Smad and PKA are located in a signaling pathway upstream of PAI-1 [23, 24]. PTX is known as an antiplatelet agent that attenuates TGF-*β* induced collagen synthesis [25] and a nonspecific phosphodiesterases (PDE) inhibitor [17] that subsequently activates protein kinase A (PKA). Therefore, PTX treatment could modulate both TGF-*β*/Smad and PKA. Irradiation of the lung resulted in increased expression of TGF-*β*/Smad-3, a pivotal pathway in RILF [24, 26, 27], but no effect of PTX treatment on this pathway was observed. On the other hand, PTX treatment restored PKA phosphorylation, which was significantly decreased in the irradiated group. We also demonstrated antifibrotic potential of PTX by modulating PKA but not TGF-*β*/Smad in irradiated lung epithelial cells. In parallel with these findings, it was demonstrated in TGF-*β*-stimulated vascular smooth muscle cells that the anticollagen synthesis effects of PTX are mediated predominantly through a cyclic adenosine 3′, 5′-monophosphate- (cAMP-) PKA effector pathway [28] that is physiologically modulated by PDE, a target molecule of PTX [29, 30]. These findings raise the possibility that PTX-induced inhibition of PAI-1 is associated with PKA activation in RILF.

In conclusion, our results provided the bases to support the concept that PTX alone could provide useful therapeutic strategies to mitigate RILF and these molecular findings suggest that the downregulatory effect of PTX on PAI-1 may be mediated by PKA despite the absence of an observable effect of PTX on TGF-*β*/Smad in RILF.
